# Two in One: Epithelioid angiomyolipoma within a classic kidney angiomyolipoma - a case report

**DOI:** 10.1186/s12882-018-0919-0

**Published:** 2018-05-30

**Authors:** Jan Tuma, Holger Moch, Gerd Stuckmann, Walter Gysel, Andreas L. Serra

**Affiliations:** 1 Ultrasound Learning Center EFSUMB, Klinik Hirslanden, Zürich, Switzerland; 2 Institut für Pathologie, Universitätsspital, Zürich, Switzerland; 3 Institut für Radiologie, Kantonsspital, Winterthur, Switzerland; 4 Stiftung für Wissenstransfer, Hefenhofen, Switzerland; 5 Klinik für Innere Medizin und Nephrologie, Klinik Hirslanden, Witellikerstrasse 40, 8032 Zürich, Switzerland

**Keywords:** Epithelioid angiomyolipoma, Contrast enhanced ultrasound, Computed tomography, Biopsy

## Abstract

**Background:**

Epithelioid angiomyolipoma is defined as potentially malignant mesenchymal neoplasm, characterized by proliferating epithelioid cells, whereas classic angiomyolipoma, composed of fat, smooth muscle cells and dysmorphic vessels, is defined as a potentially benign. The usual or classic angiomyolipoma is often found incidentally on imaging studies, relatively easily identified due to the presence of fat, in contrast to the epithelioid angiomyolipoma that can pose diagnostic challenges.

**Case presentation:**

We report a 51-year-old female patient in which an ultrasonography examination showed a solid mass close to the right renal pelvis with hypoechoic and hyperechoic areas. A differential diagnosis of atypical sinus lipomatosis, lipoma and a transitional cell carcinoma was postulated whereas in a subsequent computed tomography a classic angiomyolipoma was postulated. A re-examination by contrast enhanced ultrasound revealed a striking perfusion difference of the hypoechoic and hyperechoic areas. The hypoechoic area showed homogenous and prolonged enhancement whereas the hypoechoic area displayed a marked slower contrast material flooding and a relatively rapid wash out. The histological analysis from the biopsy of the hyperechoic area showed a classic angiomyolipoma, whereas the sample of the hypoechoic central portion revealed an epithelioid angiomyolipoma. A nephrectomy was performed because of the malignant potential of the epithelioid variant of the angiomyolipoma.

**Conclusions:**

A solid kidney mass with two sharply defined parts, one-part compatible with a classical angiomyolipoma and the other being suspected of carcinoma, is rare, but also illustrative and instructive. The combination of different imaging modalities in the work up of a solid renal mass facilitated to discriminate benign from malignant areas.

## Background

Epithelioid angiomyolipoma is defined as a potentially malignant mesenchymal neoplasm, characterized by proliferating epithelioid cells, whereas classic angiomyolipoma, composed of fat, smooth muscle cells and dysmorphic vessels, is considered benign [1]. Classic kidney angiomyolipoma appears in 0.3% of the general population and accounts for 3% of solid renal masses whereas epithelioid angiomyolipoma is very rare. Classic angiomyolipoma and epithelioid angiomyolipoma have been associated with tuberous sclerosis [2, 3].The usual or classic angiomyolipoma is often found incidentally on imaging studies, relatively easily identified due to the presence of fat, in contrast to the epithelioid angiomyolipoma that can pose diagnostic challenges as it mimics a large variety of neoplasms.

Ultrasound contrast agent consist of gas microbubbles enclosed in lipid shells. These microspheres are about the half size of a red blood cell and remain only intravascular and thus do not cross into the interstitial space whereas most computed tomography and magnetic resonance imaging agents cross the vessels [4]. Ultrasound contrast agent therefore is not excreted in the collecting system of the kidney facilitating the quantification of tumor perfusion by analyzing tumor vascular enhancement patterns [5]. Contrast-enhanced ultrasound allows to continuously acquire images creating a time-intensity curve as compared with magnetic resonance imaging or computed tomography with limited number of time points [6]. Furthermore, ultrasound contrast agents can be administrated multiple times for repeated acquisitions and exhibit no risk for nephrotoxicity, cerebral deposition or nephrogenic systemic fibrosis and lacks radiation burden.

On conventional B-mode ultrasound, classical renal angiomyolipoma most commonly present as a uniform hyperechoic lesion due to the presence of fat. The assessment of hyperechoic renal mass with computer tomography or magnetic resonance imaging can aid in diagnosing angiomyolipoma: negative attenuation on computer tomography and signal dropping on fat-suppressed magnetic resonance sequences are findings which refer to an angiomyolipoma. Applying contrast-enhanced ultrasound, renal angiomyolipomas are characterized by homogenous enhancement and a prolonged enhancement time during the corticomedullary and late phase. Xu et al. demonstrated that an early washout, a heterogeneous enhancement, and the presence of a peritumoral rim are lesions suspect for renal cell carcinoma. [7]

The evaluation of the dignity of solid renal masses on imaging is often challenging. Computer tomography, magnetic resonance imaging and ultrasound can often not accurately distinguish between benign and malignant renal masses [8, 9] whereas the combination of various examination techniques can complete each other in the work up of a patient with a renal mass.

## Case presentation

**Fig. 1:**
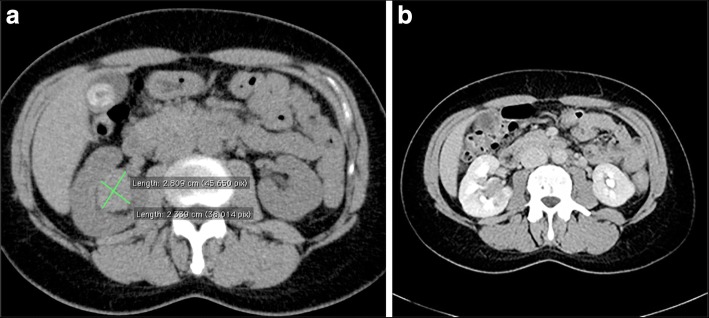
**a** Computer tomography scan without contrast material in 2008 displaying a solid mass close to the right kidney sinus of 2.81 × 2.34 cm in diameters. **b** Computer tomography scan with contrast material in 2008 displaying an early enhancement of tumor ventral margin in the arterial phase

**Fig. 2:**
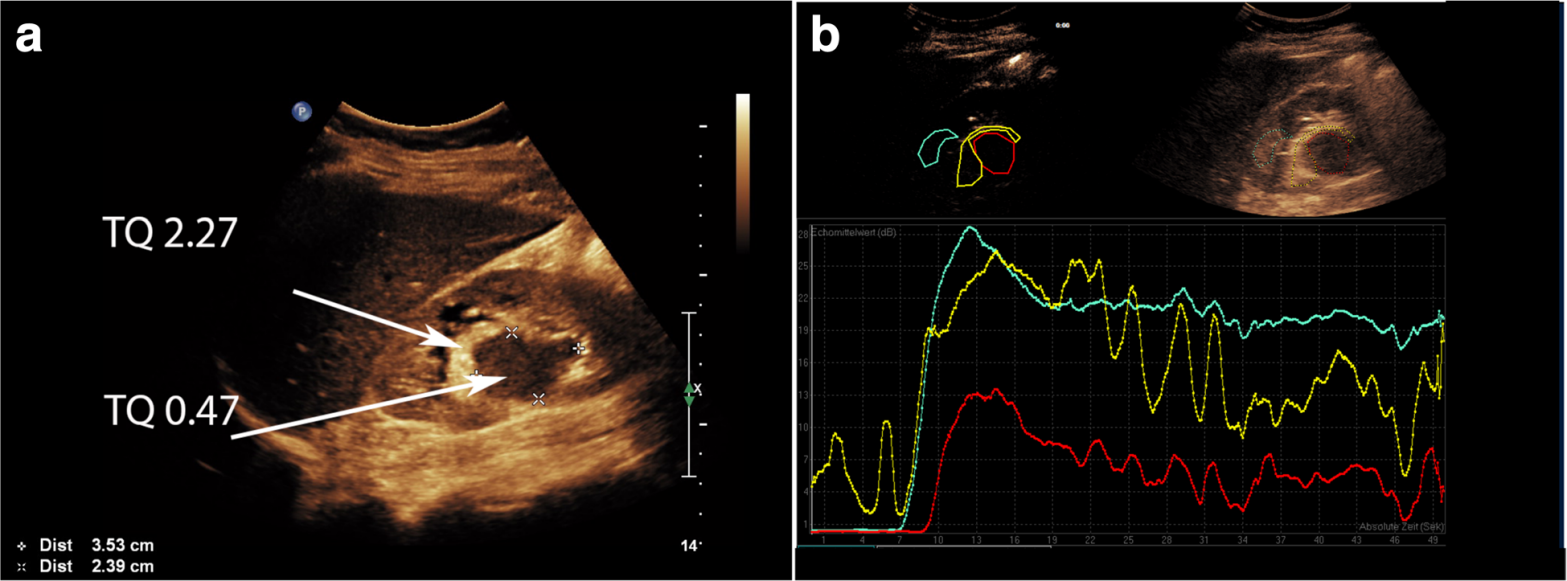
**a** B-mode ultrasound reveals two areas with different echogenicity within the tumor: Outer rim is hyperechoic with an echogenicity tumor to normal kidney cortex ratio (TQ) of 2.27 (a value larger than 2.0 is characteristic for angiomyolipoma) and an inner hypoechoic portion (TQ 0.47) [11]. **b** Time intensity curves (TIC) of the contrast enhanced ultrasound (CEUS) showing an early and strong enhancement of the hyperechoic outer rim (yellow line), similar to renal cortex (blue line), and a later and weaker enhancement of hypoechoic inner portion (red line). The strong enhancement of the outer tumor rim is clearly seen on the ventral side

**Fig. 3:**
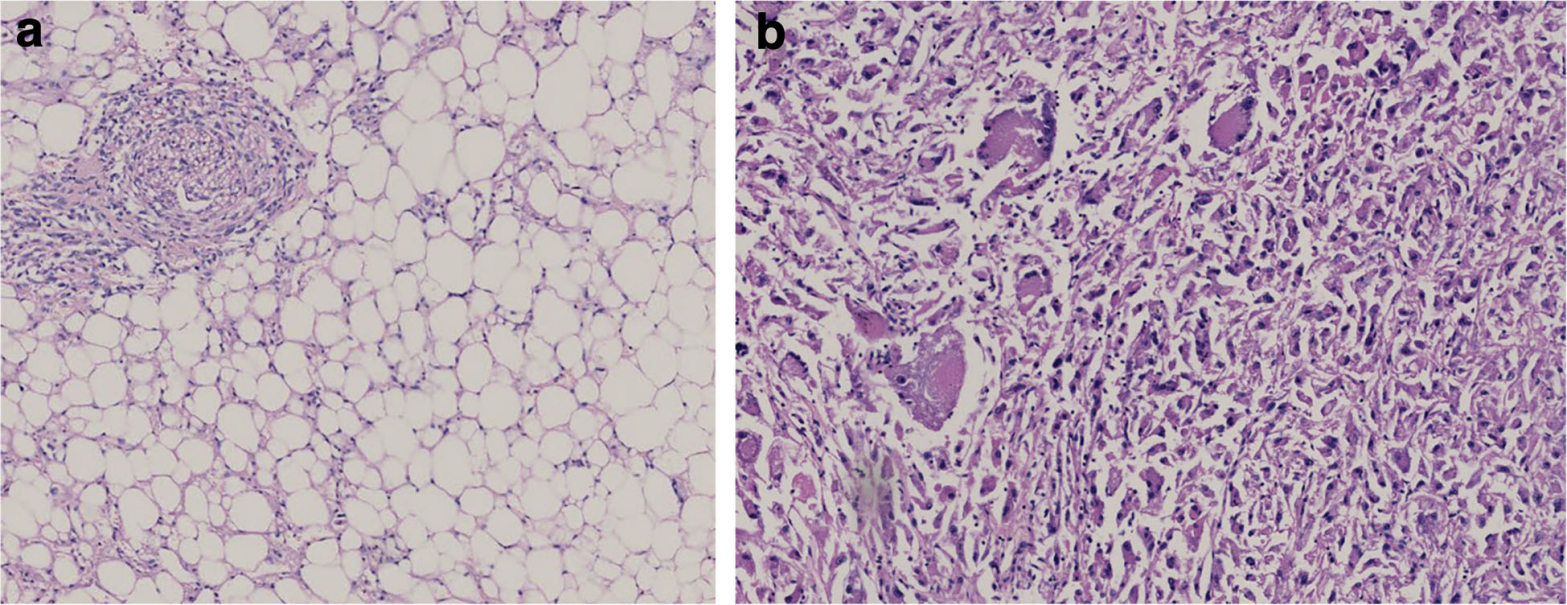
**a** Histology of the biopsy specimen taken from the hyperechoic tumor rim showing many vessels and fatty tissue confirming the diagnosis of angiomyolipoma. **b** Histology of the biopsy specimen taken from the inner hypoechoic tumor showing pleomorphic multinucleated giant cells and cells with predominant eosinophilic cytoplasm revealing an epithelioid variant of an angiomyolipoma

A 52-year-old female patient with recurrent urinary tract infections underwent abdominal ultrasound examination. The right kidney ultrasound showed a sharply restricted, hypoechoic solid renal mass of 2.5 cm diameter without posterior acoustic shadowing closely located to the renal sinus. The kidney was not congested. Atypical sinus lipomatosis, lipomas or a transitional cell carcinoma was suspected and subsequently a computer tomography scan was performed showing a homogenous mass that met the criteria for a classic angiomyolipoma (Fig. 1). A computed tomography scan repeated 2 years later demonstrated a possible tumor growth to 3 cm in diameters. Another 2 years later, an ultrasound examination revealed two different areas of the solid mass: a hyperechoic outer rim (echogenicity tumor to normal kidney cortex ratio (TQ) of 2.27) and inner hypoechoic portion (TQ of 0.47.) as displayed in Fig. 2a. Echogenicity was measured according to prior reported method [10–12]. A contrast enhanced ultrasound was performed displaying a distinct different perfusion pattern of these two areas. In the hyperechoic peripheral area, we noticed a strong perfusion that started nearly simultaneously with the renal cortex and a marked slower contrast material flooding with a relatively rapid wash out in the hypoechoic portion of the tumor (Fig. 2b). The finding of the contrast enhanced ultrasound of the hyperechoic part of the tumor was in line with a classic angiomyolipoma whereas the dignity of the hypoechoic part remained unclear. A target biopsy of both parts was performed. The histological examination showed a classic angiomyolipoma in the biopsy specimen of the outer rim (Fig. 3a) and an epithelioid angiomyolipoma in the biopsy specimen of the center part (Fig. 3b). Immunohistochemistry showed strong positivity for melanocytic markers and smooth muscle markers, confirming the diagnosis of epithelioid angiomyolipoma. A subsequent nephrectomy was performed confirming the diagnosis.

## Discussion and conclusions

The incidence of occasional detection of renal masses has increased with the wider application of various imaging modalities [13, 14]. The individual imaging methods alone can often not determine the dignity of kidney tumors. The majority of benign kidney tumors consist of oncocytomas and angiomyolipomas [15]. Benign tumors do not require intervention unless they cause the patient discomfort. The usual or classic angiomyolipoma is relatively easily identified by applying current imaging modalities. The high fat content in classic angiomyolipomas causes the tumors to appear very echogenic in the B-mode of ultrasound and in computed tomography an intensity of − 20 Hounsfield units is recorded. In our case, a differential diagnosis of atypical sinus lipomatosis, lipoma and a transitional cell carcinoma was postulated whereas based on a subsequent computed tomography a classic angiomyolipoma was postulated.

However, a re-examination by contrast enhanced ultrasound revealed a striking perfusion difference of the hypoechoic and hyperechoic areas. Prior studies suggest that contrast-enhanced ultrasound is a valuable method in distinguishing angiomyolipoma from renal cell carcinoma [7, 16–18]. The outer rim of the renal mass described in our patient displayed the classical features of an angiomyolipoma: negative attenuation on the computer tomography scan, hyperechoic appearance on the B-Mode and homogenous and prolonged enhancement of the ultrasound contrast agent. Classic angiomyolipomas are composed of fat, smooth muscle cells and dysmorphic vessels and thus the ultrasound contrast agent, which does not trespass the vessels, enhanced this part of the tumor rapidly and homogenously. The inner portion of the renal mass was hypoechoic and showed a low enhanced by the ultrasound contrast material due to the absence of fat and a considerable low number of vessels as shown by the histological analysis. Epithelioid variant of angiomyolipoma (epithelioid angiomyolipoma) is a rare tumor defined as a potentially malignant mesenchymal neoplasm that has been described first by Martignoni as a distinct variant of angiomyolipoma [19]. Epithelioid angiomyolipoma is characterized by the presence of plump and spindled epithelioid cells with varying degrees of nuclear atypia and pleomorphic multinucleated cells are often also present [1, 20]. According to the current WHO classification, tumors with 80% and more epithelioid cells are considered epithelioid angiomyolipoma [21]. Kidney epithelioid angiomyolipoma have been reported often as a single case or small case series of 20 to 41 patients and although solid evidence of the dignity is spare, epithelioid angiomyolipoma are considered as a malignant neoplasm [22–27].

Our case for the first time displays the perfusion pattern assessed by contrast enhanced ultrasound of classic angiomyolipoma and epithelioid angiomyolipoma in a single solid renal mass. Two distinct areas of the tumor were identified by contrast enhanced ultrasound: A hyperechoic part with strong perfusion and a hypoechoic part with weak perfusion and fast contrast material wash out. The result of the targeted biopsy revealed the diagnosis of an epithelioid angiomyolipoma embedded within a classic angiomyolipoma. A nephrectomy was performed because of the malignant potential of the epithelioid variant of the angiomyolipoma. In prior reported cases, diagnosis was made often after tumor resection or in removed kidneys. Resection of angiomyolipoma is considered when the tumor diameter exceeds 4 cm or hemorrhage has occurred [28–30], although potential life threatening bleeding has been reported to occur also at lower diameter [31].

Epithelioid angiomyolipoma can pose diagnostic challenges [32–34]. Indeed, our report of a 51-year-old male patient with a solid mass close to the right renal pelvis demonstrates that a combination of different imaging modalities including a diligent contrast enhanced ultrasound imaging studies with ultrasound-guided target biopsies enabled diagnosis making and treatment.

## Abbreviations

WHO: World Health Organization
